# Comparative analysis of gut microbiota and host phenotypic characteristics across enterotype-like clusters in cynomolgus and rhesus macaques

**DOI:** 10.3389/fmicb.2026.1775757

**Published:** 2026-03-12

**Authors:** Jeonghwa Choi, Kyuyoung Shim, Gyu-Seo Bae, Eunsu Jeon, Eun-Ha Hwang, Green Kim, Seung Ho Baek, Jung Joo Hong, Dae-Soo Kim, Seok-Hwan Kim, Bon-Sang Koo

**Affiliations:** 1 National Primate Research Center, Korea Research Institute of Bioscience and Biotechnology, Cheongju, Chungbuk, Republic of Korea; 2 KRIBB School of Bioscience, Korea University of Science & Technology (UST), Daejeon, Republic of Korea; 3 Digital Biotech Innovation Center, Korea Research Institute of Bioscience and Biotechnology, Cheongju, Chungbuk, Republic of Korea; 4 Department of Surgery, College of Medicine, Chungnam National University, Daejeon, Republic of Korea

**Keywords:** core microbiome, enterotype, microbiome, non-human primate, phenotype

## Abstract

Microbiome has been increasingly recognized for its close association with host physiology and diseases. Due to their close genetic relatedness to humans and standardized environmental conditions, captive macaque species serve as the most evolutionarily comparable preclinical animal models for studying human microbiome research. However, the characterization of gut microbiota and host phenotypic traits within each enterotype-like cluster of macaque species remains poorly understood. We analyzed microbiome characteristics and host metadata within enterotype-like clusters of cynomolgus and rhesus macaques housed in the same facility but with different origins of birth. At the phylum level, *Bacteroidota, Firmicutes, Spirochaetota*, and *Proteobacteria* were predominantly observed in both species. Except for *Fibrobacterota* at the phylum level, no significant interspecies differences were observed in bacterial composition or alpha diversity across taxonomic levels. Based on a prevalence threshold of 90%, cynomolgus macaques were found to share 12.4% of genera, while rhesus macaques shared 18.2%. Based on the relative abundance patterns of the genera *Prevotella 9, Rikenellaceae RC9 gut group*, and *Treponema*, the fecal microbiome of cynomolgus macaques was classified into three enterotype-like clusters (cluster 1, cluster 2, and cluster 3) whereas that of rhesus macaques was classified into two enterotype-like clusters. Using linear mixed-effects models, we identified species-specific associations between enterotype-like clusters and host phenotypes. In cynomolgus macaques, clustering was primarily associated with hematological and selected biochemical parameters, whereas in rhesus macaques, enterotype-like clusters were limited to body weight and hemoglobin. Despite a standardized diet and shared environments, distinct clusters and pronounced microbial individuality associated with birthplace suggest that early-life colonization is a key determinant of long-term gut microbiome structure and host phenotypes in captive primates. Also, identifying enterotype-like clusters in NHPs prior to analysis is essential for accurate and relevant human microbiome modeling, since each cluster may correspond to distinct human enterotypes and phenotypic traits.

## 1 Introduction

More than 100 trillion microorganisms inhabit various anatomical sites, including the gastrointestinal tract, nasal passages, oropharynx, vagina, and skin. Their composition varies across these sites due to strong niche specialization (Ma et al., 2024). The gastrointestinal tract contains the highest density and diversity of microorganisms, including bacteria, yeasts, viruses, and fungi, the numbers of which are comparable to or even greater than those of the host's cells. Following birth, the gut is colonized by external microbes, leading to the establishment of a relatively stable microbiome that confers colonization resistance against pathogenic invaders in adulthood (Bäckhed et al., 2015; Wampach et al., 2017). However, the established microbiome exhibits high variability, arising from the dynamic interplay among host, microbial, and environmental factors (Procházková et al., 2024; Wu et al., 2011; Dong and Gupta, 2019). Even within the same species, the stabilized microbiome demonstrates a high degree of microbial individuality (Zhou et al., 2024).

The gut microbiome is increasingly recognized for its role in modulating host physiology and influencing the progression of various diseases. It has been linked to obesity, neurological, cardiovascular, gastrointestinal, diabetic, infectious, and other metabolic disorders (Carding et al., 2015; Krishnan et al., 2015). These associations have been extensively investigated through human clinical studies and preclinical animal models including mice, honeybees, zebrafish and non-human primates (NHPs) (Douglas, 2019; Park and Im, 2020; Kang et al., 2024). However, microbiome research faces limitations depending on whether it is based on studies involving humans or preclinical animal models. In human studies, personalized microbiomes have been consistently observed among individuals. Moreover, it is challenging to control long-term environmental factors that influence the microbiome throughout the experimental period (Lee and Chang, 2021). Therefore, longitudinal evaluation should be necessary for human individuals but collecting samples before the onset of diseases is highly difficult (Lee and Chang, 2021; Lyu et al., 2023). These constraints pose significant challenges to clarifying the microbiome's role in human health and diseases.

Studies utilizing various preclinical animals, such as mice, for microbiome research face limitations in translating findings to humans due to substantial differences of host phylogenetics in genetics, anatomy, physiology and immunology. It has recently been revealed that the gut microbiome mirrors the host phylogeny across related host species, a phenomenon referred to as *phylosymbiosis* (Amato et al., 2019; Brooks et al., 2016). Therefore, selecting experimental animal species that are closely related to humans is critical for advancing human microbiome research, particularly in clarifying the correlations between the microbiome and diseases. Old World monkeys represent the preclinical animal species with the highest evolutionary similarity to humans among those legally used in biomedical research (Zhang et al., 2014). Among them, rhesus and cynomolgus macaques are predominantly bred in China which was one of the biggest producers of NHPs worldwide (Zhang et al., 2014). According to the USDA Animal Welfare Report, approximately 60-75% of NHPs used in research are rhesus macaques (Rogers et al., 2002). Compared to other animal species, these macaques exhibit a gut microbiome that more closely resembles that of humans (Li et al., 2018). Notably, captive macaque models display gut microbiome profiles that closely resemble those of humans. The conditions of captivity can significantly modify their indigenous microbial communities, frequently leading to a shift from *Prevotella*-dominated to *Bacteroides*-dominated profiles (Li et al., 2018; Clayton et al., 2016).

Microbiome research utilizing NHPs have been increasingly reported in recent years (Clayton et al., 2016; Kang et al., 2024; Li et al., 2018; Cui et al., 2019). Given the genetic heterogeneity observed in conventional preclinical animal models and the environmental variability inherent to human clinical studies, captive NHPs serve as appropriate and well-controlled models for studying the human microbiome. However, the characterization of gut microbiota and host phenotypic traits within each enterotype-like cluster remains poorly understood, especially in cynomolgus and rhesus macaques, which are among the most commonly used NHP models in experimental and biomedical studies. In this study, we conducted a comparative analysis of the gut microbiome and host metadata between indoor-housed cynomolgus and rhesus macaques maintained under identical environmental conditions for at least 1 year. Furthermore, we performed enterotype-like clustering and investigated the microbiome profile and host phenotypic characteristics within each cluster.

## 2 Materials and methods

### 2.1 Animal information

This study included 81 healthy adult macaques aged 5-12 years, consisting of 50 cynomolgus monkeys (*Macaca fascicularis*) and 31 rhesus monkeys (*Macaca mulatta*). The animals were originally sourced from China, Cambodia, and the Republic of Korea (Primate Resource Center, Korea Research Institute of Bioscience and Biotechnology), and were subsequently maintained for at least 1 year in indoor cages at the National Primate Research Center (NPRC) of the Korea Research Institute of Bioscience and Biotechnology (Supplementary Figure 1A). Age categories were defined according to previously established developmental stages in cynomolgus macaques (Tan et al., 2025; Li et al., 2023; Chen et al., 2023). All animals included in this study fell within the adolescence (4-6 years) and youth (7-13 years) stages; therefore, age-related analyses were restricted to these two groups (Supplementary Figure 1A). The facility was automatically controlled to maintain a temperature of 24 ± 2 °C, relative humidity of 50 ± 5%, light intensity of 150_−_300 lux, ventilation at 10-20 air changes per hour, and a 12 h light/12-h dark cycle. All animals were fed a commercial monkey diet (2050 Teklad Global 20% Protein Primate Diet; Harlan, Envigo, UK), which contains 20% crude protein, 5.4% fat, 8.1% crude fiber, and 40.1% carbohydrates. Animals were monitored daily by institutional breeders, and veterinary evaluations were performed periodically by institutional primate veterinary specialists. All animals tested negative for enteric pathogens and parasites, including *Campylobacter jejuni, Clostridium difficile, Salmonella* spp., *Shigella* spp., and *Yersinia enterocolitica*. Housing and care were conducted in accordance with the guidelines outlined in *The Guide for the Care and Use of Laboratory Animals* (Institute for Laboratory Animal Research, 2010) (Council, 2010).

### 2.2 Physical measurement, hematology and blood chemistry

After an overnight fast, body weight was measured, and blood samples were obtained from the femoral veins of animals anesthetized with ketamine sodium (10 mg/kg). Collected blood was transferred into two types of collection tubes: ethylenediaminetetraacetic acid (EDTA-K_2_) tubes for hematological analysis and sodium heparin tubes for blood chemistry analysis. Plasma was separated by centrifugation at 1,600 × g for 15 min. Hematological parameters were measured using a HEMAVET 950FS analyzer (Drew Scientific Ltd., USA), and plasma biochemical parameters were analyzed using a Dri-Chem 7000i biochemistry analyzer (Fujifilm, Japan). The interpretation of hematological and biochemical parameters was performed based on reference values previously established and reported by our institution (Koo et al., 2019).

### 2.3 DNA extraction and 16S rRNA sequencing

Fresh fecal samples were aseptically collected within 2 hours of defecation and transported to the laboratory on ice. Total DNA was extracted using the QIAamp Fast DNA Stool Mini Kit (Qiagen, Valencia, USA). PCR amplification targeted the V3–V4 hypervariable regions of the 16S rRNA gene using the primer set 341F and 805R. Dual-index barcoding was performed using Illumina Nextera i5 and i7 index primers according to the manufacturer's protocol (Amplicon et al., 2013). Negative controls (no-template controls) were included during PCR amplification to monitor potential contamination. Amplicons were purified using the CleanPCR system (CleanNA, The Netherlands), and the quality and size of the purified products were assessed with a DNA 7,500 chip on a Bioanalyzer 2,100 (Agilent, USA). Sequencing was carried out on the Illumina MiSeq platform (Illumina, USA). All sequencing procedures were conducted by CJ Bioscience Inc. (Republic of Korea).

### 2.4 Meta genomic data processing

All data processing procedures were performed using the Microbial Genomics Module in CLC Genomics Workbench version 23.0.4 (CLC Bio, Qiagen, Germany). Raw sequencing reads were imported in FASTQ format, and low-quality sequences were trimmed and filtered according to the following criteria: sequence length < 200 bp, presence of ambiguous and adapter bases, and a quality score < 0.05. High-quality paired-end reads were subsequently merged, and amplicon sequence variants (ASVs) were inferred through DADA2-based denoising procedures including removal of chimeric sequences and the filtering of reads with insufficient overlap or low abundance. Taxonomic classification was conducted using the SILVA 138.1 reference database with a 99% similarity threshold.

### 2.5 Microbiome data analysis and visualization

Alpha diversity metrics, including Shannon diversity and observed richness, were calculated using the *microbiome* R package (Lahti and Shetty, 2018). Beta diversity was assessed by calculating Bray–Curtis dissimilarities and performing Principal Coordinate Analysis (PCoA) using the *vegan* package (version 2.6-4) (Oksanen et al., 2019). The optimal number of clusters (*k*) was determined by jointly considering multiple internal validation metrics, including the average Silhouette width and the Calinski–Harabasz (CH) index, together with bootstrap-based cluster stability assessed using the Jaccard similarity index (500 iterations). Enterotype-like clustering was identified based on genus-level relative abundance profiles using Partitioning Around Medoids (PAM). Differential abundance of bacterial genera was assessed using the *ANCOMBC2* package. Data visualization was performed in R using the *ggplot2* ecosystem, including alluvial plots, chord diagrams, and hierarchical heatmaps (Brunson, 2020; Villanueva and Chen, 2019).

### 2.6 Statistical significance

Statistical significance between groups was evaluated using non-parametric tests. Comparisons between two groups were performed using the Mann-Whitney U test, whereas comparisons among three or more groups were conducted using the Kruskal–Wallis test. When significant differences were detected by the Kruskal–Wallis test, *post hoc* pairwise comparisons were performed using Dunn's test with Bonferroni correction, and adjusted *p*-values were reported. For analyses incorporating covariates, linear mixed-effects models (LMMs) were used to evaluate differences in host metadata among enterotype-like clusters, with country of origin included as a covariate to account for geographic variation. Analysis of covariance (ANCOVA) was performed as a supplementary approach. *Post hoc* pairwise comparisons were performed using Tukey's honestly significant difference (HSD) test following ANCOVA, and Tukey-adjusted estimated marginal means (emmeans) following LMM analyses. To assess the independent effect of enterotype-like clustering while accounting for country of origin, a marginal (Type III) PERMANOVA was performed using the adonis2 function in the vegan R package (Bray-Curtis dissimilarity, 999 permutations). The specific statistical test used for each analysis is indicated in the corresponding figure legends or Results section.

## 3 Results

### 3.1 Fecal microbiome characteristics of cynomolgus and rhesus macaques

**Figure 1:**
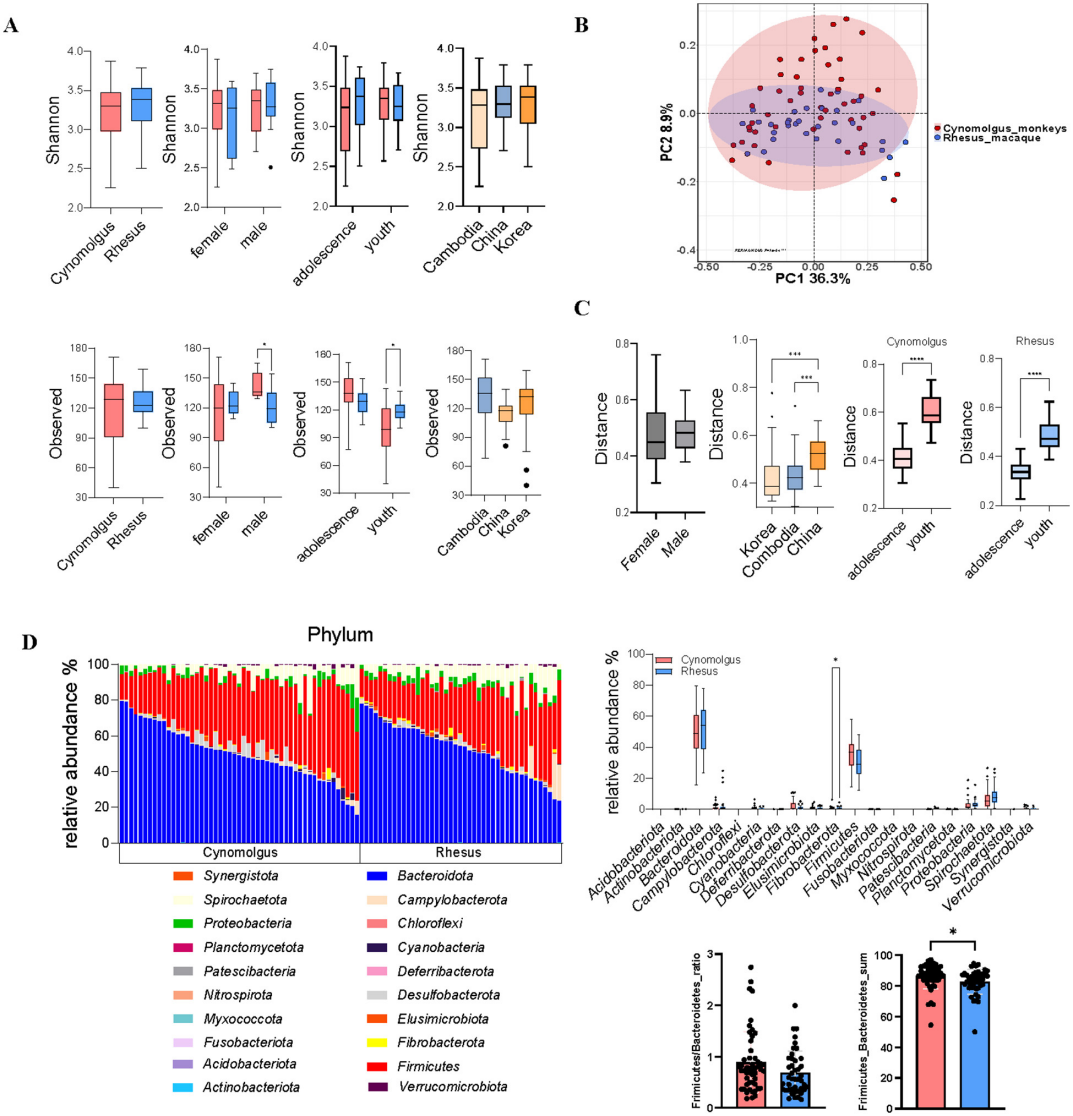
Comparative analysis of gut microbiome characteristics between cynomolgus and rhesus macaques. Multifaceted comparison of gut microbiota between cynomolgus (red) and rhesus (blue) macaques. **(A)** Alpha diversity metrics (Shannon index and observed richness) in cynomolgus and rhesus macaques according to age group, sex, and country of origin. **(B)** Principal coordinate analysis (PCoA) of gut microbial beta-diversity in cynomolgus and rhesus macaques. **(C)** Bray–Curtis beta-diversity distances stratified by age group, sex, and country of origin. **(D)** Relative abundance of major bacterial phyla in cynomolgus and rhesus macaques. Adolescence (4–6 years) and youth (7–13 years) to assess age-related effects. Statistical comparisons using the Mann–Whitney U test, depending on data normality (*p* < 0.05 was considered significant).

**Figure 2:**
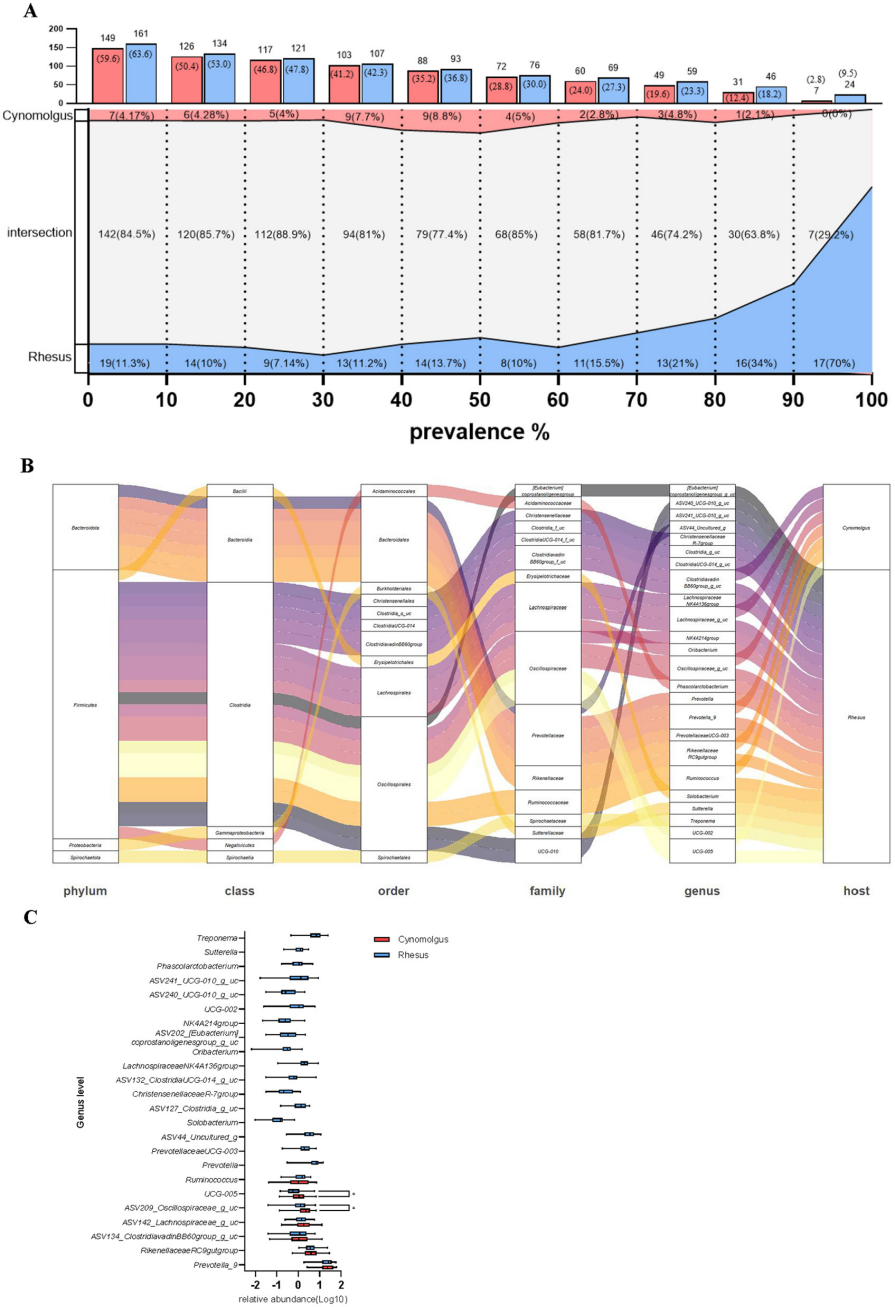
Comparison of shared bacterial taxa and their relative abundance between cynomolgus and rhesus macaques. **(A)** The number and proportion of shared bacterial taxa between two species across different prevalence thresholds. **(B)** Alluvial diagram showing the taxonomic distribution (from phylum to genus level) of bacterial taxa with 100% prevalence in each species. **(C)** Genus-level relative abundance of bacterial taxa with 100% prevalence in cynomolgus and rhesus macaques.

The V3–V4 region of the 16S rRNA gene was successfully sequenced from fecal samples of animals, all fed identical diets and reared under standardized conditions at the NPRC facility. Alpha diversity did not show significant variation based on animal species, sex, or country of origin (Figure 1A). PCoA-based beta diversity analysis revealed that while the two animal species shared a portion of their fecal microbiota, each also harbored species-specific microbial communities (Figure 1B). In both species, no significant differences in the gut microbiome were observed between sexes, whereas variations were detected based on the animals' origin and age (Figure 1C). At the phylum level, the combined relative abundance of *Firmicutes* and *Bacteroidetes* was significantly higher in cynomolgus macaques compared to rhesus macaques. The relative abundance of *Fibrobacterota* was higher in rhesus macaques than in cynomolgus macaques (Figure 1D). No significant differences in bacterial composition between the two species were identified at other taxonomic levels (Supplementary Figure 2B). We successfully conducted an analysis of the core microbiome proportions at different prevalence thresholds within each species, and identified the shared bacterial taxa and their corresponding proportions between the two species (Figures 2A, B; Supplementary Figures 3A, B). A higher relative abundance of two genera within the family *Oscillospiraceae* was observed in cynomolgus macaques compared to rhesus macaques (Figure 2C).

### 3.2 Species-specific host metadata characteristics

Host metadata, including age, sex, species, country of origin, body weight, hematological parameters, and blood chemistry values, were successfully determined in all animals. Rhesus macaques exhibited significantly higher body weights compared to cynomolgus macaques (Supplementary Figure 1B). In the blood chemistry analysis, levels of alkaline phosphatase (ALP), blood urea nitrogen (BUN) and albumin (ALB) were higher in rhesus macaques, whereas inorganic phosphorus (IP) level was lower compared to cynomolgus macaques (Supplementary Figure 1C). Hematological evaluation revealed that cynomolgus macaques had a higher relative abundance of white blood cell (WBC), lymphocytes (LY), monocyte (MO), red blood cell (RBC) and platelets (PLT). Cynomolgus macaques exhibited significantly higher hematocrit (HCT) and lower mean corpuscular hemoglobin concentration (MCHC) than rhesus macaques (Supplementary Figure 1D).

### 3.3 Enterotype-like clusters

**Figure 3:**
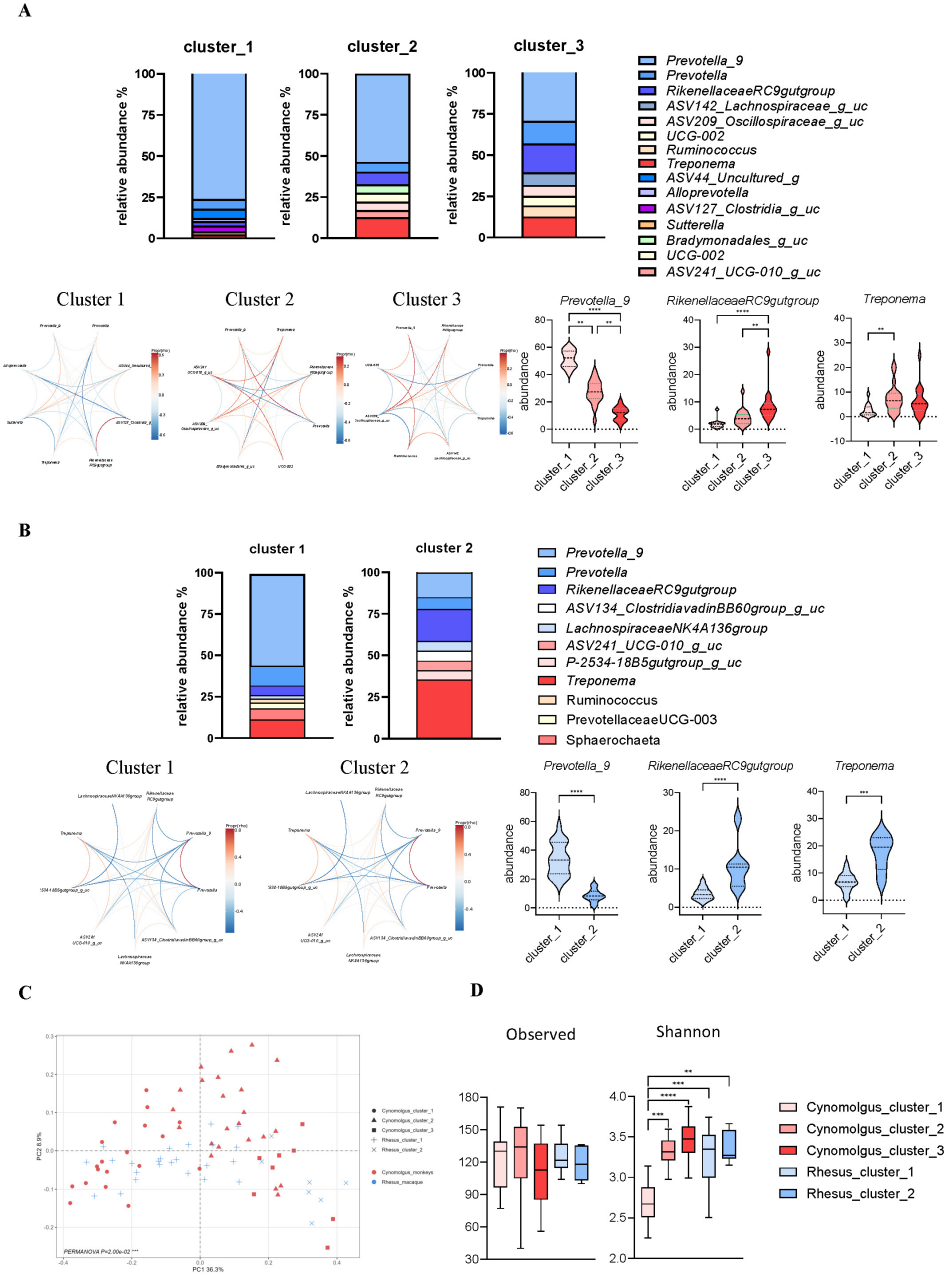
Enterotype-like clustering of cynomolgus and rhesus macaques. **(A, B)** Genus-level relative abundance profiles showing enterotype-like clustering in cynomolgus **(A)** and rhesus **(B)** macaques. Clusters were identified using PAM and evaluated by PERMANOVA (adonis2, 999 permutations), including marginal (Type III) models adjusting for country of origin. Optimal cluster number was determined using Silhouette width, CH index, and bootstrap Jaccard stability. Co-occurrence networks of bacteria genera within each enterotype in both species. Abundance of main bacterial contributor for clustering of enterotype within each species. **(C)** Beta diversity (Bray–Curtis dissimilarity) among enterotypes in cynomolgus and rhesus macaques. **(D)** Alpha diversity (Shannon index and observed richness) among enterotypes in cynomolgus and rhesus macaques.

**Figure 4:**
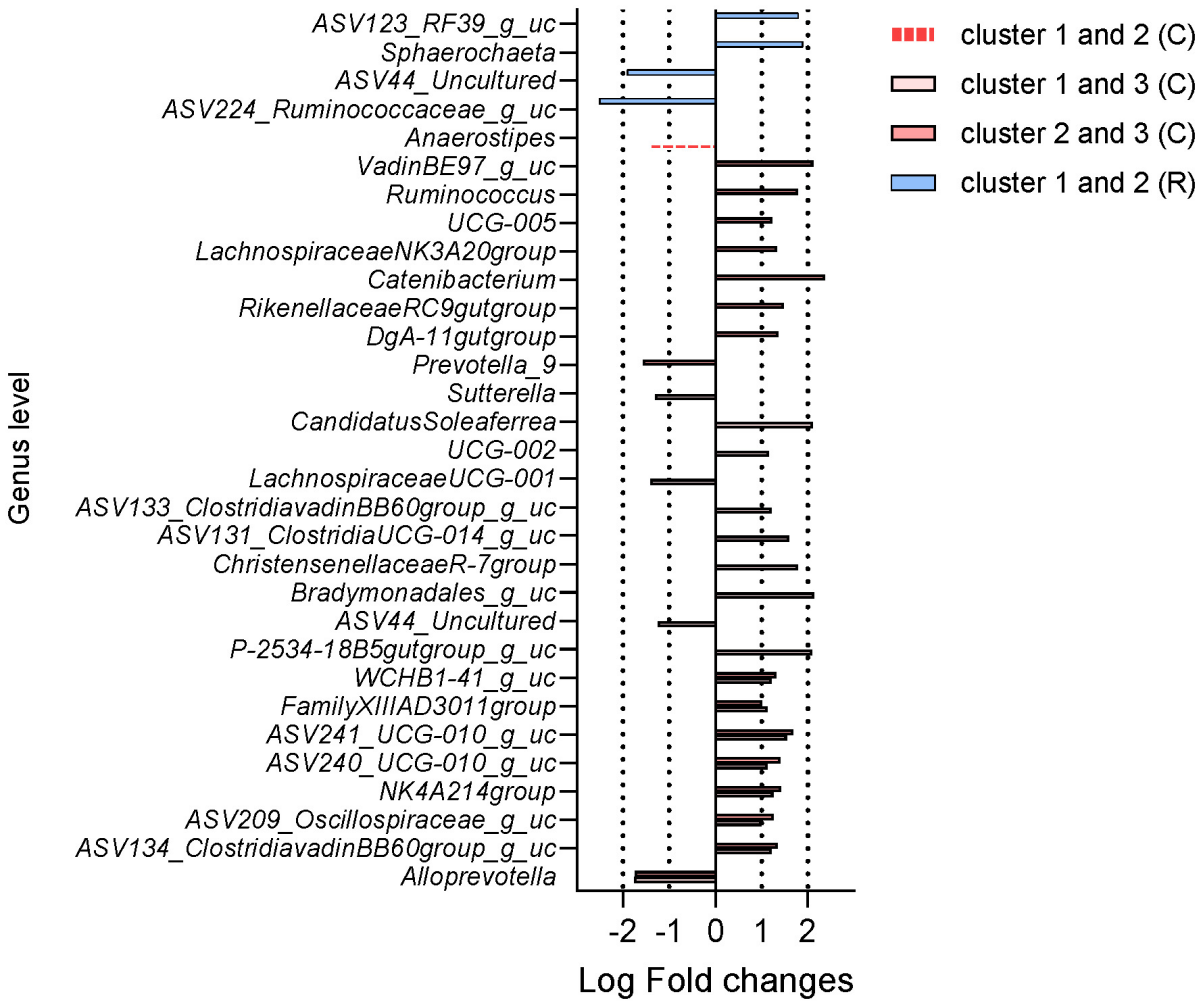
Differential abundance of bacterial genera among enterotype-like clusters in cynomolgus and rhesus macaques. Genus-level differential abundance among enterotype-like clusters identified using ANCOMBC2 with FDR correction (*q* < 0.05, |LFC| > 1.0), with comparisons labeled as C (cynomolgus macaques) and R (rhesus macaques).

Cluster validation showed species-specific patterns: cynomolgus macaques supported *k* = 3 based on Silhouette (0.18) and CH index (0.16), with robust bootstrap stability (mean Jaccard = 0.91), whereas rhesus macaques showed optimal separation and maximal stability at *k* = 2 (Silhouette = 0.34; CH = 18.12; mean Jaccard = 0.83). The fecal microbiomes of cynomolgus and rhesus macaques were classified into enterotype-like clusters based on the relative abundances of the dominant genera, including *Prevotella 9, Rikenellaceae RC9 gut group*, and *Treponema*. In cynomolgus macaques, three enterotype-like clusters (Clusters 1, 2 and 3) were identified, whereas the rhesus macaque microbiome was optimally partitioned into two enterotype-like clusters. In cynomolgus macaques, enterotype explained 34.5% of the variation in microbial composition (*R*^2^ = 0.3448, *p* = 0.001), whereas country of origin accounted for 17.5% (*R*^2^ = 0.1751, *p* = 0.001). After adjusting for origin, enterotype remained significantly associated with microbial composition (marginal *R*^2^ = 0.2512, *p* = 0.001). Similarly, in rhesus macaques, enterotype explained 34.8% of the variance (*R*^2^ = 0.3481, *p* = 0.001), and this association persisted after adjustment for origin (marginal *R*^2^ = 0.2484, *p* = 0.001). In contrast, the independent effect of origin was attenuated after accounting for enterotype (*R*^2^ = 0.0423, *p* = 0.107). In cynomolgus macaques, cluster 1 was characterized by a high relative abundance of *Prevotella 9 and* low levels of *Rikenellaceae RC9 gut group* and *Treponema* (Figure 3A). Enterotype-like cluster 2 exhibited intermediate relative abundances of *Prevotella 9* and the *Rikenellaceae RC9 gut group*, while the abundance of *Treponema* was higher than that observed in cluster 1. Cluster 3 was characterized by the highest relative abundance of the *Rikenellaceae RC9 gut group* and the lowest abundance of *Prevotella_9* among the three clusters (Figure 3A). In rhesus macaques, two enterotype-like clusters were identified. Cluster 1 was dominated by *Prevotella 9*, whereas Cluster 2 was characterized by higher relative abundances of the *Rikenellaceae RC9 gut group* and *Treponema*. This two-cluster structure in rhesus macaques corresponds to a consolidation of the cynomolgus macaque Cluster 1 (*Prevotella*-dominant) and the combined features of Clusters 2 and 3, which were enriched in *Rikenellaceae RC9 gut group* and *Treponema* (Figure 3B). PCoA-based beta diversity analysis revealed that Cluster 1 showed partial overlap between the two macaque species, while Clusters 2 and 3 exhibited a separated, species-specific distribution (Figure 3C). Alpha diversity, as measured by the Shannon index, differed significantly among clusters in cynomolgus macaques (Figure 3D). The distribution of individuals within each cluster varied according to their country of origin (Supplementary Figure 2B). Several microbial taxa that differed significantly among the clusters were identified within each species (Figure 4; Supplementary Figures 4A, B).

### 3.4 Cluster-specific host metadata characteristics

**Figure 5:**
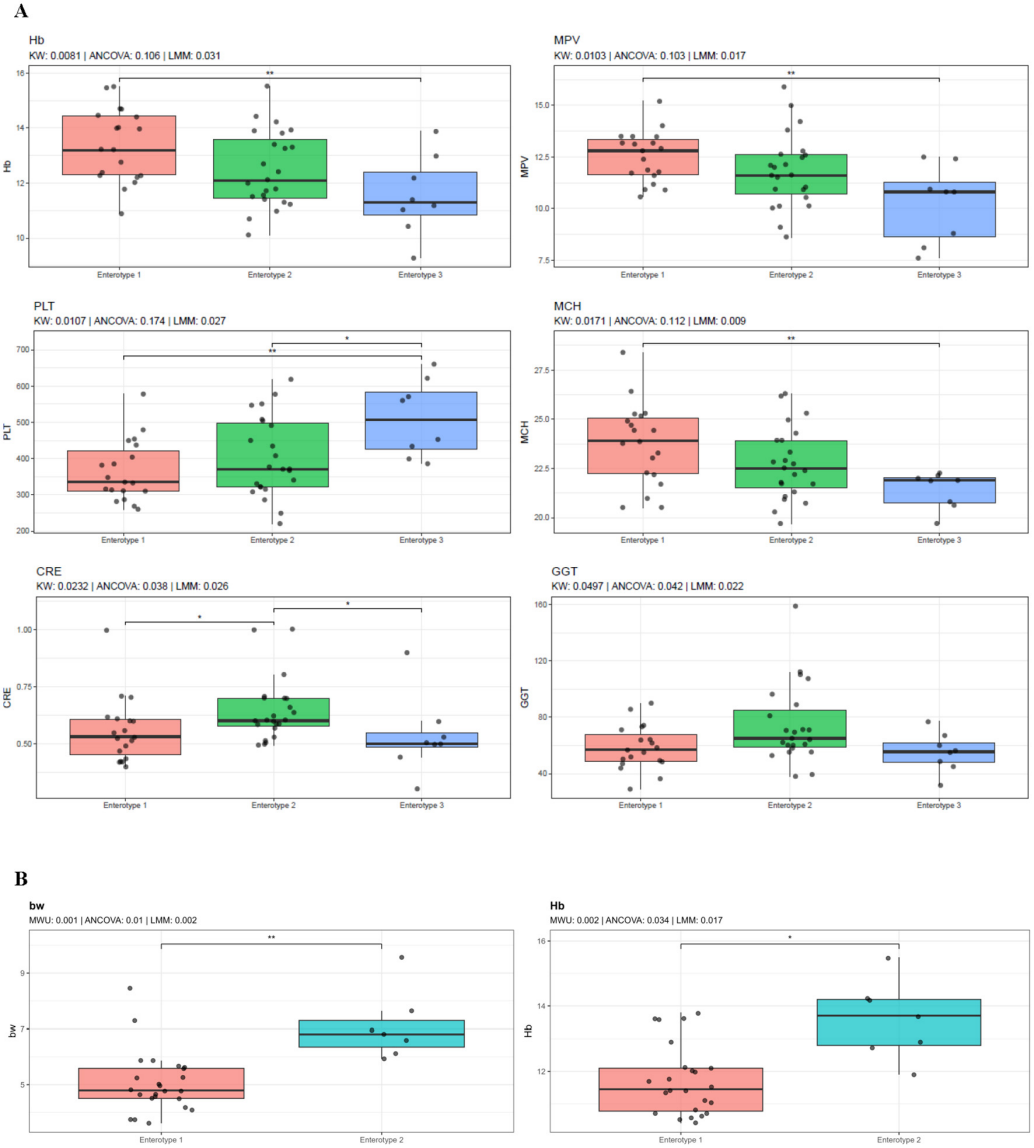
Host phenotypic characteristics among enterotype-like clusters in cynomolgus and rhesus macaques. Comparison of body weight, blood chemistry and hematological parameters among enterotype-like clusters in cynomolgus **(A)** and rhesus **(B)** macaques. For three-group comparisons (cynomolgus), differences were assessed using the Kruskal–Wallis test with Dunn's *post hoc* test, and for two-group comparisons (rhesus), the Mann–Whitney U test was applied. ANCOVA and linear mixed-effects models (LMMs) adjusting for country of origin were additionally performed. Only variables significant in both non-parametric tests and LMMs (*p* < 0.05) are shown. *P*-values are displayed above each panel. Asterisks indicate significant pairwise differences (*p < 0.05, **p < 0.01, ***p < 0.001).

In both cynomolgus and rhesus macaques, differences in body weight, hematological parameters, and selected blood biochemical values were observed across clusters; however, the specific variables showing differences varied between the two species (Figure 5). After adjusting for country of origin using LMMs, enterotype-like clusters remained significantly associated with distinct host phenotypic traits in both species (Figure 5). In cynomolgus macaques, cluster-associated differences were primarily observed in hematological and biochemical parameters, including hemoglobin (Hb), mean platelet volume (MPV), PLT, mean corpuscular hemoglobin (MCH), creatinine (CRE), and gamma-glutamyl transferase (GGT) and mean corpuscular volume (MCV). In rhesus macaques, significant cluster-associated differences were observed for body weight (BW) and Hb levels (Figure 5B).

## 4 Discussion

The microbiome has been increasingly recognized for its close association with host physiology and diseases (Carding et al., 2015; Krishnan et al., 2015). Consequently, the selection of appropriate animal models for microbiome research should reflect human-related characteristics in terms of microbial composition, physiological traits, and pathological responses. Among the legally available preclinical animal models worldwide, Old World NHPs exhibit the highest phylogenetic similarity to humans. In this study, we conducted an in-depth comparative analysis of gut microbiota composition and host-associated metadata, focusing on interspecies differences between two of the most commonly used captive Old World NHPs models, cynomolgus macaques and rhesus macaques, as well as on variations among enterotype-like clusters identified within each species.

At the phylum level, *Bacteroidota, Firmicutes, Spirochaetota*, and *Proteobacteria* were predominantly observed in both species (Figure 1). In another facility where cynomolgus and rhesus macaques were co-housed under identical conditions, *Bacteroidota, Firmicutes*, and *Proteobacteria* were dominant. In this facility, *Firmicutes* were more abundant in rhesus macaques, while *Bacteroidota* and *Proteobacteria* were enriched in cynomolgus macaques. At the genus level, *Prevotella* and *Treponema* were dominant in cynomolgus macaques, whereas *Ruminococcaceae, Oscillospira*, and *Faecalibacterium* were more abundant in rhesus macaques. Higher alpha diversity was also observed in cynomolgus macaques in that facility (Cui et al., 2019). In contrast, our study found no significant interspecies differences at the phylum level, except for *Fibrobacterota*, no significant differences at the genus level, and no significant differences in alpha diversity between the two species (Figure 1). The differences between our findings and a previous report may be partly explained by differences in country of origin. In our research, country of origin was a major contributor to gut microbiota variability (Figure 1C). Unlike another facility where cynomolgus and rhesus macaques were imported from distinct regions, animals in our facility originated from overlapping geographic areas, including China and Korea, which may have attenuated interspecies differences in gut microbiota composition and diversity (Supplementary Figure 1A). In previous reports, microbiome composition in captive cynomolgus and rhesus macaques shows considerable variation among facilities (Sawaswong et al., 2021; Li et al., 2018; Adriansjach et al., 2020). These findings highlight that while the microbiome composition appears similar in the same species, differences in relative abundance are evident. Therefore, establishing group-specific reference microbiome data is essential in NHP studies.

The core microbiome has been defined using a wide range of prevalence thresholds, typically spanning from 30 to 100%, depending on the study design and research objectives (Tan et al., 2023; Risely, 2020). Based on occurrence, previous studies have shown that genera constituting the core microbiome, defined by a prevalence threshold of 90%, account for only approximately 1-10% of all detected genera in the human gut microbiome (Neu et al., 2021; Qu et al., 2025; Falony et al., 2016). This indicates that most genera are individually variable, and that the core microbiome is highly limited. Based on a prevalence threshold of 90%, cynomolgus macaques were found to share 12.4% of genera, while rhesus macaques shared 18.2% in this report (Figure 2). Across matched occurrence thresholds, humans shared 102, 62, and 18 genera at 50%, 80%, and 100% prevalence, respectively, whereas cynomolgus macaques shared 88, 49, and 7 genera and rhesus macaques shared 93, 59, and 24 genera at the corresponding thresholds (Ruiz-Ruiz et al., 2026). These findings indicate that cross-species patterns are comparable across prevalence thresholds, with only a modest increase observed in macaques under the more stringent 90% criterion. Overall, cross-species patterns remain largely similar, even after more than 1 year of standardized feeding. Also, the individual relative proportions of *Firmicutes* and *Bacteroidota* at the phylum level varied considerably among the animals. This diversity was found to be primarily associated with age and country of origin (Figure 1C). This indicates that early-life microbial colonization, shaped by the geographic origin of the host, may have a lasting impact, maintaining its distinct composition throughout life despite prolonged exposure to standardized diet and environmental conditions, which may also have contributed to a moderate increase in core microbiome composition compared to that observed in humans (Palmer et al., 2007). These findings are consistent with a previous study in rhesus macaques, which reported marked diet-associated shifts in the gut microbiota during infancy, but minimal age-related changes in adulthood (Janiak et al., 2021).

In this study, enterotype-like clusters refer to dataset-specific microbial community groupings derived from unsupervised clustering of gut microbiota composition. This terminology has been previously adopted in NHPs and other animal microbiome studies to distinguish such clusters from canonical human enterotypes (Li et al., 2018; Wang et al., 2014; Arumugam et al., 2011). Enterotype-like clustering suggested three groups in rhesus macaques and two groups in cynomolgus macaques, which were differentiated based on the relative abundance of *Prevotella 9, Rikenellaceae RC9 gut group*, and *Treponema* (Figure 3). In another facility, three enterotype-like clusters enriched in *Prevotella, Lactobacillus*, and *Ruminococcus* were identified, with *Prevotella* and *Bacteroides* being the predominant genera in Chinese-origin cynomolgus macaques (Li et al., 2018). In wild gorillas and chimpanzees, the relative abundance of *Prevotella* increases during periods when their diet is rich in plant-based foods (Hicks et al., 2018). In humans, enterotypes are commonly classified based on dominant bacterial genera such as *Bacteroides* and *Prevotella*. *Bacteroides* is typically associated with protein-rich diets, whereas *Prevotella* is predominantly observed in populations consuming high-fiber, high-carbohydrate diets (Arumugam et al., 2011; Wu et al., 2011; Gorvitovskaia et al., 2016). *Treponema* species are predominantly observed in traditional rural populations and are rarely detected in individuals from urban areas (Schnorr et al., 2014; Obregon-Tito et al., 2015). Also, low abundance of *Treponema* is commonly observed in NHPs under high-fat dietary conditions (Prince et al., 2019). At the phylum level, *Spirochaetes* are observed exclusively in traditional rural populations and are not detected in individuals from urban areas (Obregon-Tito et al., 2015). Therefore, the identified clusters may share compositional features with microbial patterns previously described in humans. Both species could be grouped into a *Prevotella*-rich cluster (Cluster 1) and *Treponema*-enriched clusters (Clusters 2 and 3). These interpretations represent descriptive comparisons based on human-derived enterotype classification and do not imply shared biological characteristics. Rather, human enterotype frameworks serve as a reference for interpreting macaque gut microbiota clustering within this dataset. Based on this interpretation, careful selection of macaques according to microbiota clusters may help account for biological variability in primate-based preclinical microbiome studies and potentially improve translatability. However, unlike previous studies in which enterotypes were primarily classified based on differences in diet and lifestyle, the clusters identified in this study remained clearly distinguishable despite the animals being maintained under identical long-term dietary and environmental conditions.

In this study, we conducted a comparative analysis of host metadata between the two species and among clusters within each species (Figure 5 and Supplementary Figure 1). Based on LMM-adjusted data accounting for country of origin, cluster-related variation in cynomolgus macaques was primarily observed in several hematological and biochemical parameters, including platelet-related indices (PLT and MPV), erythrocyte-related parameters (Hb, MCH, and MCV), as well as liver-related (GGT) and renal-related (CRE) markers. In rhesus macaques, only BW and Hb differed across clusters. Overall, these cluster-associated differences were modest and limited to a small subset of hematological and biochemical parameters. Although the clinical relevance of these differences remains limited at this stage, the observed associations cautiously suggest the possibility that enterotype-like clusters may be linked to variation in selected host hematological and biochemical parameters. Therefore, further longitudinal investigations and functional validation studies will be required to clarify whether these cluster-associated differences reflect biologically meaningful host–microbiome interactions.

In this study, we conducted a comparative analysis of microbiome in two NHP species with different origins of country but raised under long-term standardized dietary and environmental conditions. We successfully classified enterotype-like clusters within each species based on distinct differences in key gut microbial taxa, and observed corresponding variations in host phenotypic traits across these clusters. Despite a standardized diet, shared environment, and similar genetic backgrounds, a high degree of dissimilarity in microbiome composition was observed, which was strongly associated with the animals' country of origin. These findings suggest that early-life factors linked to geographic origin may contribute to the establishment of individualized gut microbiome patterns that persist under standardized conditions. However, the present study is observational in nature and does not establish a causal relationship between gut microbiome composition and host phenotypic variation. Rather, our results generate the hypothesis that stable, individualized microbiome configurations may influence host phenotypes independently of recent dietary intake. From a translational perspective, our findings underscore the importance of identifying enterotype-like clusters in NHPs models prior to downstream analyses. Such cluster-based classification may improve the relevance and interpretability of microbiome–phenotype associations and facilitate more accurate modeling of human enterotypes and associated phenotypic traits.

## Data Availability

All 16S rRNA gene sequencing data from this study are available in the NCBI BioProject database under accession number PRJNA1275590.

## Supplementary material

Supplementary Figure 1 **(A–D)** Comparison of host phenotypic parameters between cynomolgus and rhesus macaques. Each point represents an individual animal, horizontal bars indicate mean ± standard deviation (SD). Mann-Whitney U test. *p* < 0.05 considered significant.

Supplementary Figure 2 Comparative analysis of gut microbiome characteristics in cynomolgus and rhesus macaques. **(A)** Principal Coordinates Analysis (PCoA) of microbial composition with PERMANOVA results, colored by country of origin. **(B)** Distribution of country of origin across enterotypes. **(C)** Relative bacterial abundance at the family taxonomic level.

Supplementary Figure 3 Prevalence-based taxonomic distribution of bacterial taxa in cynomolgus and rhesus macaques. **(A, B)** The distribution of bacterial taxa at each prevalence percentage in cynomolgus **(A)** and rhesus **(B)** macaques.

Supplementary Figure 4 Hierarchical clustering of gut microbial profiles in cynomolgus and rhesus macaques. Heatmap of Z-score–normalized relative abundances at the family **(A)** and genus **(B)** across individual samples. Samples are hierarchically clustered and annotated by animal species (top), country of origin, and microbiome cluster (bottom).
