# Supplementary figures and images for: Comparative analysis of gut microbiota and host phenotypic characteristics across enterotype-like clusters in cynomolgus and rhesus macaques
Source: Front Microbiol. 2026 Mar 12;17:1775757. doi: 10.3389/fmicb.2026.1775757 (PMC13017862; doi:10.3389/fmicb.2026.1775757)

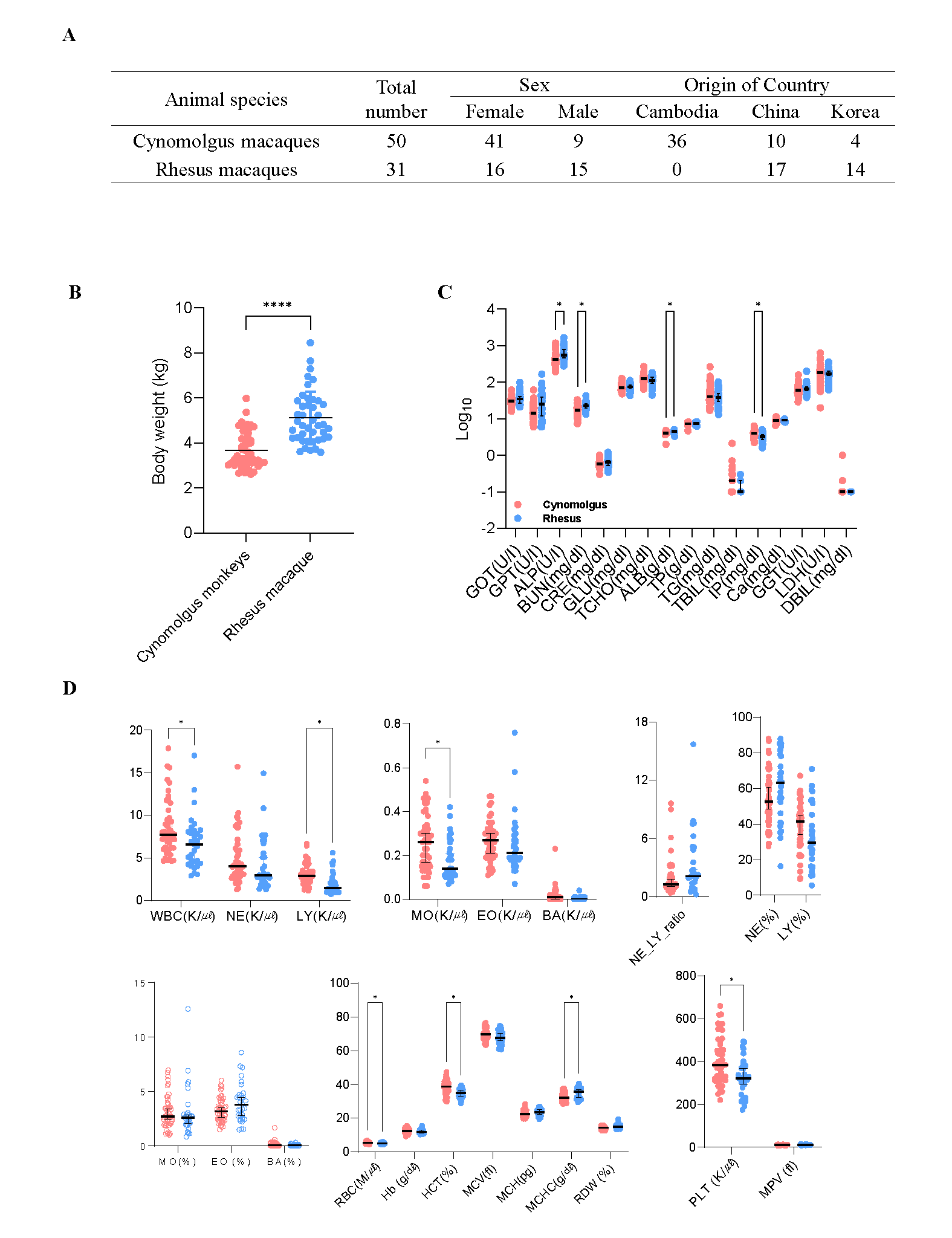

Supplement: Supplementary Figure 1 — (A–D) Comparison of host phenotypic parameters between cynomolgus and rhesus macaques. Each point represents an individual animal, horizontal bars indicate mean ± standard deviation (SD). Mann-Whitney U test. p < 0.05 considered significant. [file Image_1.tif]

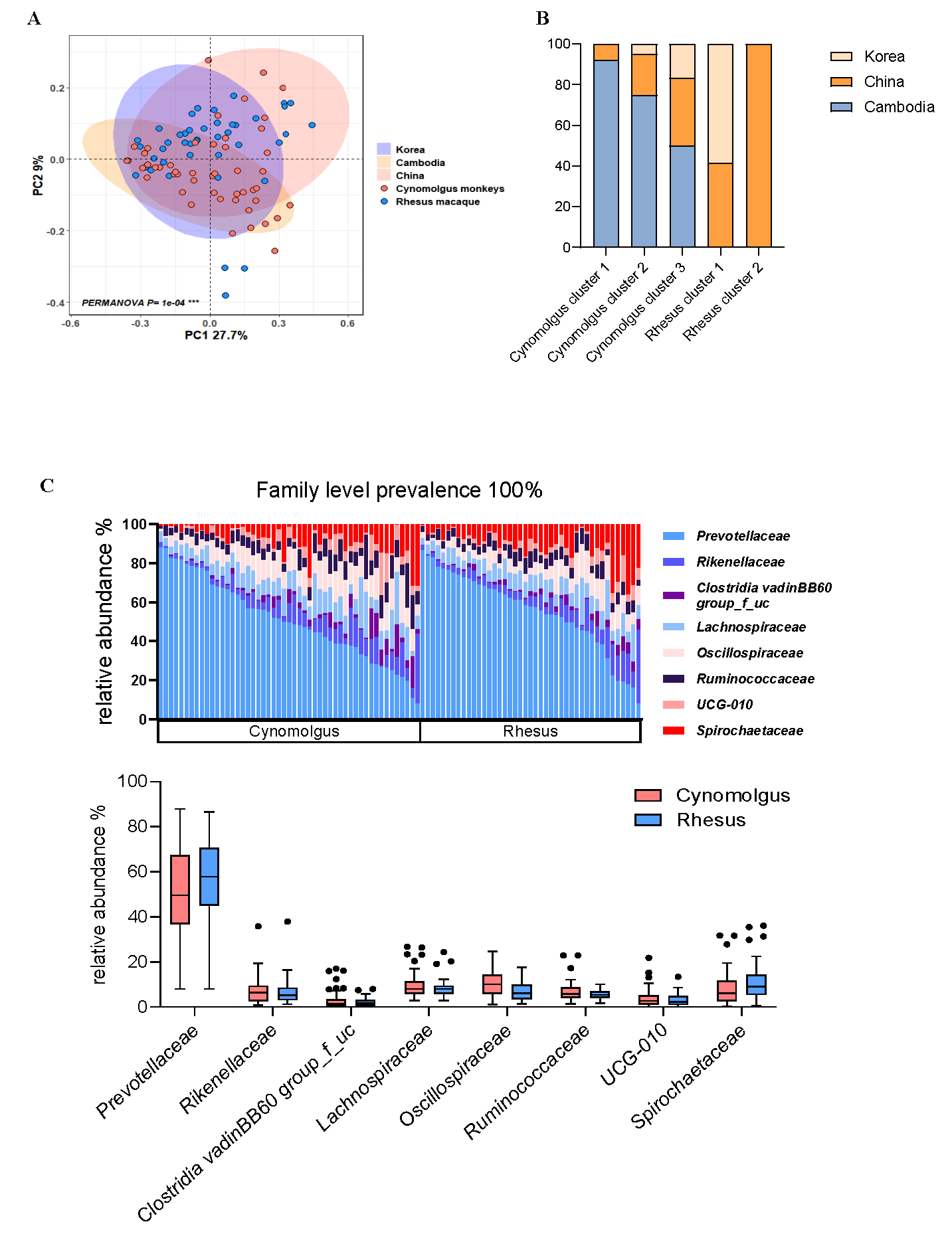

Supplement: Supplementary Figure 2 — Comparative analysis of gut microbiome characteristics in cynomolgus and rhesus macaques. (A) Principal Coordinates Analysis (PCoA) of microbial composition with PERMANOVA results, colored by country of origin. (B) Distribution of country of origin across enterotypes. (C) Relative bacterial abundance at the family taxonomic level. [file Image_2.tif]

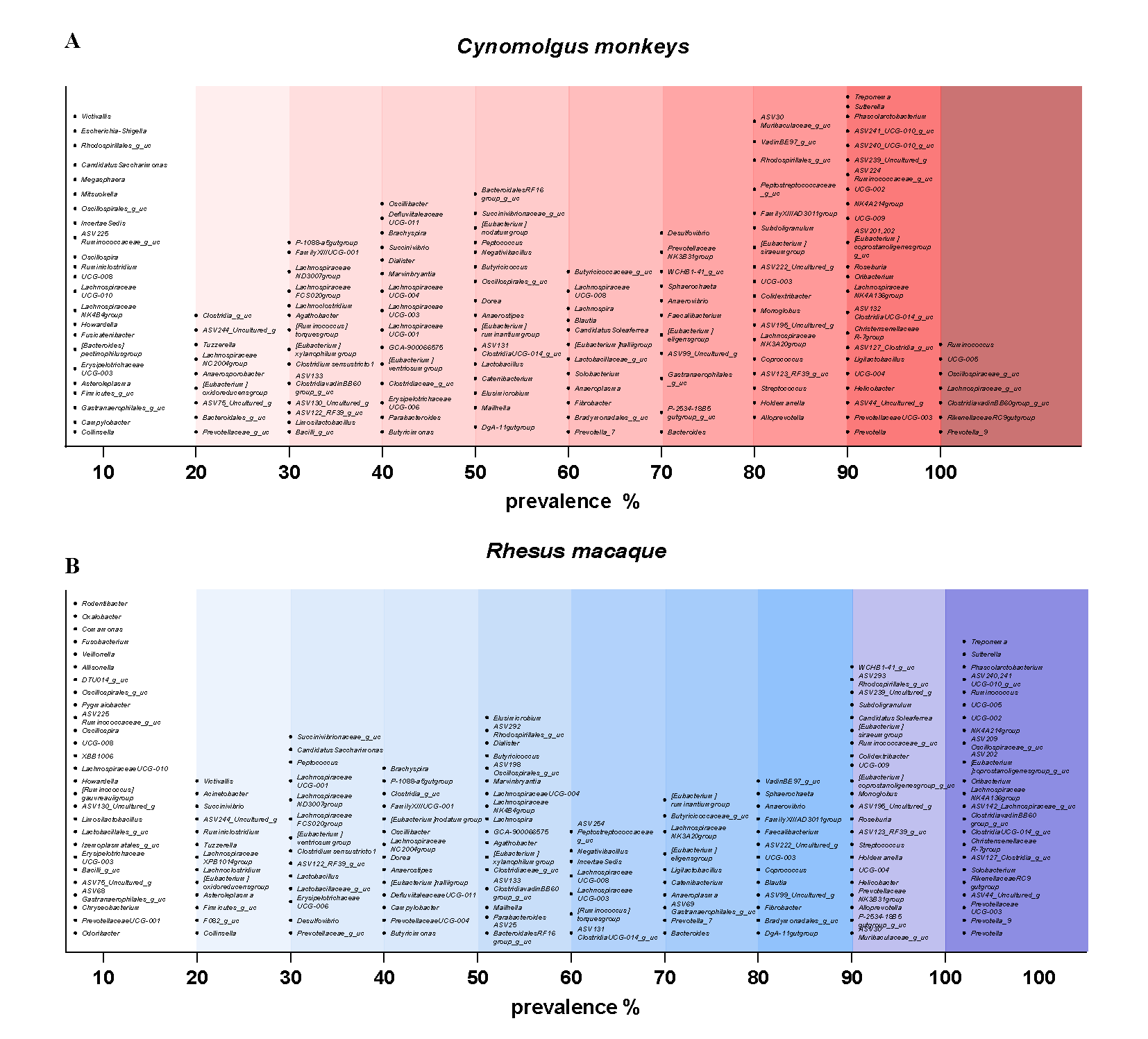

Supplement: Supplementary Figure 3 — Prevalence-based taxonomic distribution of bacterial taxa in cynomolgus and rhesus macaques. (A, B) The distribution of bacterial taxa at each prevalence percentage in cynomolgus (A) and rhesus (B) macaques. [file Image_3.tif]

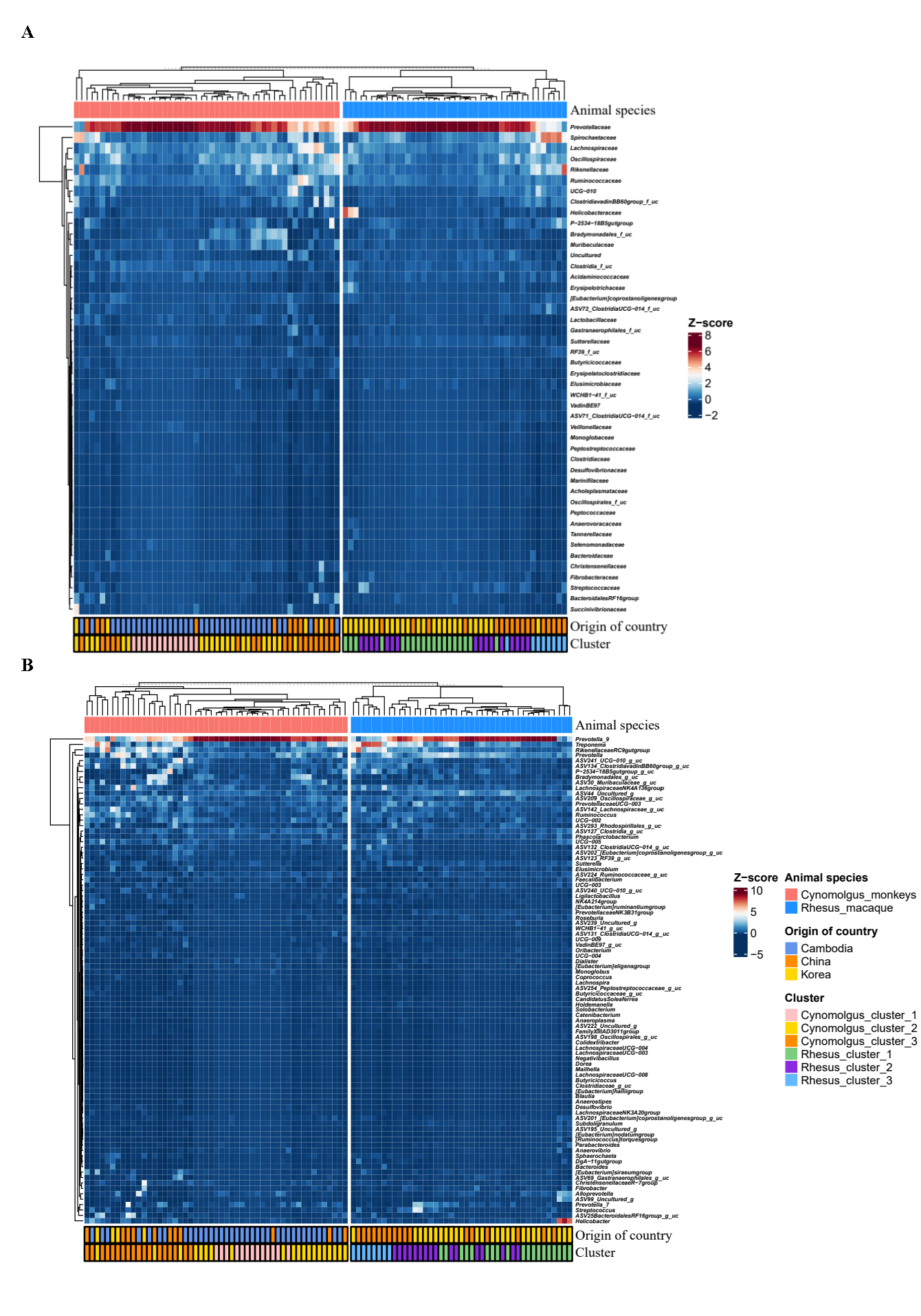

Supplement: Supplementary Figure 4 — Hierarchical clustering of gut microbial profiles in cynomolgus and rhesus macaques. Heatmap of Z-score–normalized relative abundances at the family (A) and genus (B) across individual samples. Samples are hierarchically clustered and annotated by animal species (top), country of origin, and microbiome cluster (bottom). [file Image_4.tif]
